# Combining Rice Straw Biochar With Leguminous Cover Crop as Green Manure and Mineral Fertilizer Enhances Soil Microbial Biomass and Rice Yield in South China

**DOI:** 10.3389/fpls.2022.778738

**Published:** 2022-04-25

**Authors:** Zhijian Xie, Farooq Shah, Chunhuo Zhou

**Affiliations:** ^1^College of Land Resources and Environment, Jiangxi Agricultural University, Nanchang, China; ^2^Key Innovation Center for the Integration of Industry and Education on Comprehensive Utilization of Agricultural Wastes, Prevention and Control of Agricultural Non-point Pollution of Jiangxi Province, Nanchang, China; ^3^Department of Agronomy, Abdul Wali Khan University, Mardan, Pakistan

**Keywords:** rice-straw biochar, leguminous cover crop, soil microbial biomass, rice grain yield, combining effects

## Abstract

Whether combining rice-straw biochar (RSB) with leguminous cover crop (LCC) has synergistic effects in the rice production system or not, is still unknown. Two pot experiments were conducted to systematically explore the impacts of RSB on mass decomposition and nitrogen (N) release from LCC residues after incorporation into acidic paddy soil. Similarly, the effect of combining these two factors on soil nutrient status and microbial biomasses in the rice production system was also examined. Five treatments, namely, no N fertilizer (CK), 100% N fertilizer (150 kg N ha^–1^ as N_100_), 80% N fertilizer plus RSB (N_80_B), LCC (N_80_M), and a combination of RSB with LCC (N_80_BM), were included. The results indicated that biomass decomposition and N release pattern followed a double exponential decay model such that the addition of RSB slightly stimulated the rates of both mass decomposition and N release during the initial rapid phase of decomposition. Thereafter, it notably slowed down the rates of both these parameters during the relatively slower stage of incorporating LCC residues to paddy soil during early rice season. Compared to 100% N, applying 80% N in conjunction with RSB and/or LCC residue increased grain yield and its components (i.e., effective panicles, 1,000-grain weight, and fully filled grains) that subsequently increased N accumulation and its physiological use efficiency (*PUE*_*N*_) of rice shoot. Moreover, under 20% N, applying RSB and/or LCC residue remarkably increased the soil organic matter and total N, and soil microbial populations and biomasses, while the contents of NH_4_^+^ and NO_3_^–^ were decreased in RSB-amended paddy soil (N_80_B and N_80_BM), in comparison with N_100_. Thus, combining RSB with LCC residue is a novel and promising management intervention for reducing mineral fertilizer use, improving soil fertility and rice production, and consequently minimizing the overall production cost in south China.

## Introduction

The existing pressure on agriculture to meet the global food demand is expected to further intensify as the galloping world population may roughly cross 9 billion in 2045. Rice being a staple food plays a pivotal role as it is consumed by more than half of the world’s population and about 65% of people in China (Tang and Cheng, 2019). For a hugely populated country like China, that resides more than 1.4 billion people (19% of the world’s population), another 26.9% more rice grain should be produced by 2030 in comparison with 2000 to meet its domestic need and ensure food security, if rice consumption per capita stays at the current level (Cheng et al., 2007).

The unsustainable and intensive agricultural developing pattern has generated serious soil degradation and productivity deterioration-related concerns. Excessive application of nitrogen (N) fertilizer to reddish paddy soil has further exacerbated the menace by causing considerable reactive N losses which in turn result in severe environmental problems and human health issues in China. The solution is to raise awareness of farmers on managing mineral N fertilization strategy and increasing the use of organic fertilizer, to promote sustainable development of agriculture and environment (Jiang et al., 2018). Maintaining or even improving the sustainable fertility and productivity of limited arable reddish soil is highly essential for future rice production to overcome the challenge of producing more rice grain yields with lower environmental costs.

Leguminous cover crop (LCC) that biologically fixes N from the inexhaustible pool of atmospheric N with its symbiotic rhizobia system on roots, can be grown on paddy fields which may otherwise usually be fallow after the rice harvest. LCC is regarded as an important source that adds to soil fertility by mainly providing N. It generally has considerable amounts of aboveground and underground biomass (Poeplau et al., 2015). Thus, it subsequently increases C and N fluxes into the soil and provides energy for soil organisms during rotation, thereby contributing to improve organic matter, nutrient content and bacterial community in the soil (He et al., 2020). Incorporating LCC residue into paddy soil is an appropriate alternative fertilization option, which not only produces more rice yields without increasing chemical fertilizers, but also consistently increases N use efficiency (Xie et al., 2016; Yang L. et al., 2019). Similarly, LCC reduces N pollution while maintaining net economic benefits for the overall ecosystem. Thus, partially substituting the conventional mineral N fertilizer with other alternatives will have less environmental impact without negatively affecting rice productivity (Cai et al., 2018; Ding et al., 2018; Yang L. et al., 2019). After incorporating the LCC residue into the soil, the peak decomposition and N mineralization of the LCC residue occur about 30 days after incorporation (Zhou et al., 2019). This time frame unfortunately under most conditions may not synchronize nutrients release with rice crop demand, forcing farmers to incorporate LCC residue about 15 days prior to transplanting rice seedlings. Therefore, it is necessary to synchronize biomass decay and nutrient release from LCC residue with crop nutrient demand for improving potential benefits of LCC to sustainable agriculture.

Rice straw, a representative agricultural residue that accounts for almost 29% of total crop straw and contains millions of tons of nutrients every year in China, is generally treated improperly. It is either combusted or littered directly, thereby leading to energy and nutrients waste as well as extensive N loss and critical environmental pollutions. Moreover, on-site incorporating rice straw directly prior to planting rice seedlings would cause adverse effects, such as competing N against crop plants, increasing greenhouse gas emission (e.g., CH_4_ and N_2_O), plant diseases, and insect pests (Jiang et al., 2019; Liu et al., 2021), thereby resulting in rice grain yields reduction. Hence, issues about effectively using rice straw are becoming more and more important as the concerns on food security and environmental protection increase in China. Biochar, a carbon-rich porous solid, is produced from various agricultural and forest wastes *via* oxygen-limited pyrolysis at a high temperature up to 700°C (Lehmann and Joseph, 2009). Recently, biochar has significantly received interests in increasing soil inorganic nutrients, reducing mean cumulative leaching of NO_3_^–^ due to the capability of retaining both anionic and cationic solutes, including NO_3_^–^ and NH_4_^+^, and improving plant growth and N use efficiency in soil-plant systems (Mandal et al., 2019). Therefore, generating biochar from rice straw after crop harvesting is considered a practical promising management strategy of crop residue wastes to avoid the on-site burning of crop residues which is one of the key issues related to air pollution and the reduction of soil microbial community and biomass. Agegnehua et al. (2017) showed that applying biochar benefits both acidic and neutral agriculture soils.

Red soil, the predominant type of soil in south China, is used for rice production. This type of soil is typically characterized by a low productivity mainly due to poor soil properties (e.g., low pH, lack of organic C, and nutrients). Consequently, it results in increasing reliance on chemical fertilizers (especially N fertilizer) to meet the growing demand for grain production (Bouwman et al., 2013). Thus, coping with the massive rice straw and the winter fallow paddy fields along with the excessive use of N fertilizer (leads to severe N losses) are some of the key obstacles of rice-based cropping systems for farmers in south China are confronted with. Combining rice-straw biochar (RSB) with LCC residue in a rice-based cropping system can be a novel and viable management intervention that can effectively allow recycling organic materials and tackle the abovementioned problems. As a result, it may help in improving N utilization efficiency and increasing rice yield. A previous study conducted by Kimani et al. (2021) has suggested that poultry litter biochar application in combination with chemical fertilizer and/or *Azolla* as green manure is a promising approach to improve rice productivity, reduce N use efficiency, and minimize application of chemical fertilizer, ultimately leading to a reduced agricultural pollution and production cost. However, the effects of RSB on biomass decomposition and N release from incorporated LCC residues as well as their co-incorporation on rice yield and N use efficiency in reddish paddy soil are poorly understood. Hence, we hypothesized that (i) RSB may affect the mass decomposition and N release from LCC residue after incorporation to paddy soil, (ii) co-incorporation of RSB and LCC residue will ameliorate soil properties (e.g., inorganic N, microbial population, and biomasses), (iii) their combined application may increase yield and N use efficiency in the rice-based cropping system.

## Materials and Methods

### Experimental Site, Climate, and Soil

The incubation experiment was conducted at the agroecological park of Jiangxi Agricultural University (28°45′48.3′′ N, 115°50′6.5′′ E). The experimental region has a subtropical monsoon climate, characterized by heavy rain from April to June and seasonal drought from September to December. The average annual temperature is 17.8°C, and the average annual rainfall is 1545.9 mm. The annual sunshine is 1603.4 h. The mean frost-free period lasts for 276 days. The experimental soil is silt-clay derived from Quaternary red soil. The top 0–20 cm soil was collected from a paddy field, air-dried at room temperature, and then passed through a 2-mm sieve prior to laboratory analysis. Initial properties of soil were as follows: pH 5.17 (1:2.5, soil: water), soil organic carbon (SOC) 18.7 g kg^–1^, total nitrogen (TN) 1.09 g kg^–1^, available N 86.4 mg kg^–1^, extractable phosphorus (P) 12.8 mg kg^–1^, available potassium (K) 107.8 mg kg^–1^, and contents of clay, silt, and sand were 15.7, 51.4, and 32.9%, respectively.

### Leguminous Cover Crop and Biochar

The LCC used in this study was Chinese milk vetch (*Astragalus sinicus* L.). Fresh LCC shoot was harvested at the full-bloom stage and chopped into about 3–5 cm pieces in length. Roots of the LCC were deliberately not included in the trials as a previous study conducted by Pan et al. (2011) has indicated that N accumulation in LCC root was 6.53 kg N ha^–1^, which was equivalent to 4.35% of the recommended application rate of N (150 kg ha^–1^). Meanwhile, we also conducted another pot experiment prior to this experiment and observed that the N accumulation in LCC roots was just 2.50–3.12 kg ha^–1^, which was equivalent to 1.67–2.08% of the recommended application rate of N at the experimental site (150 kg N ha^–1^). Hence, it was assumed that the addition of LCC roots would not result in a significant effect under these experimental conditions and was, therefore, not included in the soil. Another justification for not adding roots of the leguminous crop to the soil was that extraction of roots from a soil and then adding it to a crop are quite hectic and would not be practically possible, and was hence not included in the experiment. A subsample of fresh LCC shoot was taken for chemical analysis, which contained 405.2 g C kg^–1^, 32.6 g N kg^–1^, 3.45 g P kg^–1^, and 24.5 g K kg^–1^. The moisture content was 90.9%, and the C/N ratio was 12.4. The rice (*Oryza sativa* L., cv. Zhongjiazao 17) seed was provided by the School of Agronomy, Jiangxi Agricultural University, China.

The biochar was made from rice straw which was previously dried at 105°C for 24 h and milled to <2 mm. The milled rice straw was placed into ceramic crucibles and covered with close-fitting lids. Then, the straw in the ceramic crucibles was pyrolyzed under a limited oxygen supply in a muffle furnace (12D-16, Taisite Instrument Co., Ltd., Tianjina, China). The pyrolysis temperature was raised to 450°C at a rate of ∼ 20°C min^–1^ and then was kept for 3 h (Chun et al., 2004). The RSB was cooled overnight to room temperature and ground to pass through a 0.25 mm sieve. The characteristics of RSB were tested as follows: pH 9.41 (1:100, weight:volume), total organic carbon (TOC) 537.97 g kg^–1^, TN 6.13 g kg^–1^, total P (TP) 1.99 g kg^–1^, total K (TK) 27.15 g kg^–1^, specific area 242.24 m^2^ g^–1^, pore volume 0.14 cm^3^ g^–1^, and pore diameter 2.66 nm.

### Experimental Design and Management

#### Experiment 1: Soil Fertility, N Uptake, and Rice Yield

A pot experiment was conducted that included five treatments with four replications, i.e., (i) control (CK, no N fertilizer, RSB, and LCC); (ii) 150 kg N ha^–1^ (N_100_); (iii) 80% N applied in conjunction with RSB (N_80_B), (iv) 80% N applied in conjunction with LCC residue (N_80_M), and (v) 80% N applied in conjunction with RSB and LCC (N_80_BM), respectively.

The rice seed was sown in a pot (20 cm in diameter and 25 cm in height) containing 7.5 kg air-dried soil (prepared as described above) that was used for sowing rice seed. The recommended rates of chemical fertilizers to early rice season were as follows: 150 kg N ha^–1^ {also equivalent to 0.10 g N kg^–1^ soil^–1^ [urea, 46.4% N], 0.05 g P_2_O_5_kg^–1^ soil^–1^ [superphosphate, 12.0% P_2_O_5_], and 0.08 g K_2_O kg^–1^ soil^–1^ [potassium chloride (KCl), 60% K_2_O]}. The N_100_ treatment received N fertilizer at a rate of 100% recommended N rate, while 80% N treatments received N fertilizer at a rate of 80% recommended N rate. RSB and fresh LCC shoot were applied at a rate of 8.9 g kg^–1^ soil^–1^ and 10.0 g kg^–1^ soil^–1^, respectively, for the following early rice season. All fertilizers, RSB, and fresh LCC shoot were uniformly mixed with the soil in each pot during the last 10 days of April. Tap water was used to flood the pots thereafter.

Two rice seedlings (25 days old) were transplanted into each plastic pot on 25th April. During the growing period of rice plants, the soil water layer was maintained at 3 cm depth by monitoring every day unless the soil naturally dried 20 days prior to the harvest of rice plants. At harvest maturity, all rice plants were harvested during the middle 10 days of July, and grains were manually separated from the straw of rice plants. Furthermore, no additional fertilizer was applied through the rice season in this study.

#### Experiment 2: Mass Decomposition and N Release From Leguminous Cover Crop Residue After Incorporation Into Paddy Soil

To study the effects of RSB on the biomass decomposition and N release from LCC shoot residue, a nylon mesh bag method was introduced into experiment 2. This experiment had two treatments (N_80_M and N_80_BM), the same as those in experiment 1 with the exception that fresh LCC residue (3–5 cm in length) was enclosed in nylon mesh bags (mesh size 38 μm, bag size 15 cm × 10 cm) and was buried below the soil surface at 10 cm depth in continuously rice-cropped pots (*n* = 80) before 10 days of rice seedlings transplant. Rice planting and management were the same as described in experiment 1, with four replications per treatment.

### Sampling and Chemical Analysis

In experiment 1, soil samples from all pots were collected for the analysis of mineral N (NH_4_^+^ and NO_3_^–^) content at the maturity stage of rice with a soil corer (∅ 1.5 cm). In each pot, 5 soil cores were randomly collected and mixed thoroughly inside a plastic bucket to form individual bulked pot soil samples. After removing visible roots and stones, the soil samples were divided into two parts. One was sieved <0.149 mm, labeled, and then stored in plastic bags for testing the soil NH_4_^+^ and NO_3_^–^ contents according to the method described previously.

The TOC and TN were measured by TOC/TN analyzer (multi N/C^®^ 3100, Germany). Soil mineral N (i.e., NH_4_^+^ and NO_3_^–^) was extracted with 2 M KCl at a 5:1 ratio (KCl: soil, v/weight), and NH_4_^+^ and NO_3_^–^ concentrations in the filtrate were analyzed by a continuous flow analyzer (AA3, Germany).

The soil microbial biomass of carbon (SMBC) and soil microbial biomass of N (SMBN) were determined by the fumigation extraction method (Brookes et al., 1985; Vance et al., 1987). In brief, fresh soil (equivalent to 25 g oven-dry weight) was weighed into 250 ml glass bottles. Three unfumigated samples from each treatment were immediately extracted with 100 ml 0.5 M K_2_SO_4_ on a rotary shaker at 220 rpm for 30 min, and then filtered through Whatman qualitative filter paper (Chantigny et al., 2006). Other three aliquots of the same samples were fumigated with alcohol-free chloroform for 24 h at 25°C. Excess chloroform was removed by repeated evacuation, and then the samples were extracted and filtered, as described previously. Soil filtrates were stored at −20°C prior to analysis for SMBC and SMBN. Organic carbon in the filtrate was determined with a TOC analyzer (Phoenix-8000, American). Total soluble N in the filtrate was followed by alkaline persulfate oxidation and measured by dual-wavelength ultraviolet spectrophotometry (Norman et al., 1985; Cabrera and Beare, 1993). Aliquots from the un-fumigated filtrates were filtered at <0.45 μm with filter membrane and used for determining soluble organic C and N using the methods described previously. Both NH_4_^+^ and NO_3_^–^ contents of the un-fumigated samples were determined with a continuous flow analyzer (AA3, Germany).

Rice plants in each pot were harvested at the maturity stage and separated into two parts (i.e., straw and grain). Plant samples were weighed after de-enzyming (105°C, 30 min) and oven-drying (70°C, 24 h) to constant weight. All the dried plant samples were ground and sieved through <0.25 mm before laboratory analysis. TN in rice plant samples was determined by sulfuric acid digestion and Kjeldahl distillation. At physiological maturity, grains were separated from the straw.

In experiment 2, the LCC residues in mesh bags were collected at 0, 5, 10, 15, 20, 25, 30, 35, 50, 65, and 91 days after installation. Residues of LCC were carefully washed with tap water, oven-dried at 70°C for 24 h, and then weighed and milled <0.149 mm. Total C and N contents of LCC residue samples were analyzed using an elemental analyzer (Vario Macro Cube, Elementar Inc., Germany).

### Calculations and Data Analysis

The percentage of dry matter weight (DMW) N remaining of LCC residue at a given time for each experimental unit was calculated from the following equation:


(1) $$X_{r}\left(\%\right)=\frac{X_{t}}{X_{0}}\times 100$$

where *X*_*r*_ is the percentage of DMW or N remaining of LCC residue, *X*_*t*_ is the DMW or N mass at each sampling time *t*, and *X*_*0*_ is the initial DMW or N mass of LCC residue at 0 days.

To describe the decaying trend of LCC residue and N release, the DMW and N remaining of LCC residue were regressed over time (t) by using the first-order double exponential decay model from the following equation:


(2) $$y=\text{a}e^{-k_{1}t}+be^{-k_{2}t}$$

where *y* is the DMW or N remaining of LCC residue at the time *t*, *k*_*1*_ and *k*_*2*_ are the DMW decay or N release rates, and *a* and *b* are the constants.

TN accumulation (*TNA*) in top rice is the sum of N content of rice shoot.


(3) $$TNA\left(gplant^{-1}\right)={\text{N}}_{s}+{\text{N}}_{g}$$

where *N*_*s*_ is the N content in straw and *N*_*g*_ is the N content in grain rice plants.

Physiological efficiency of applied N fertilizer (*PE*_*N*_) is the ratio of the increased shoot biomass of rice plants to the N amount absorbed from fertilizer:


(4) $$PE_{N}\left(kgkg^{-1}\right)=\frac{Y-Y_{0}}{U-U_{0}}$$

where *Y* and *Y*_*0*_ are the shoot biomasses of rice plants with and without N fertilizer, respectively, and *U* and *U*_*0*_ are the N accumulation in rice plant shoots with and without N fertilizer, respectively.

Values obtained from different treatments were subjected to ANOVA tests. Separation of means was performed on significant ANOVA tests by Tukey’s honestly significant difference using the SAS package (version 9.3; SAS Institute Inc., Cary, NC, United States). Statistical significance was determined at *p* < 0.05 unless otherwise stated. SigmaPlot 10.0 and MS Excel were used to generate the graphs and tables, respectively.

## Results

### Mass Decomposition and N Release From Leguminous Cover Crop Residue

**FIGURE 1:**
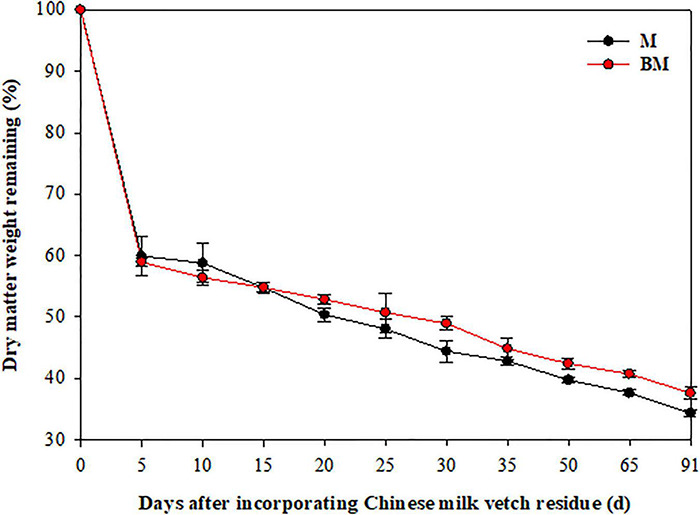
Dry matter decay pattern of leguminous crop after incorporation to reddish paddy soil. Small bars are standard errors.

**FIGURE 2:**
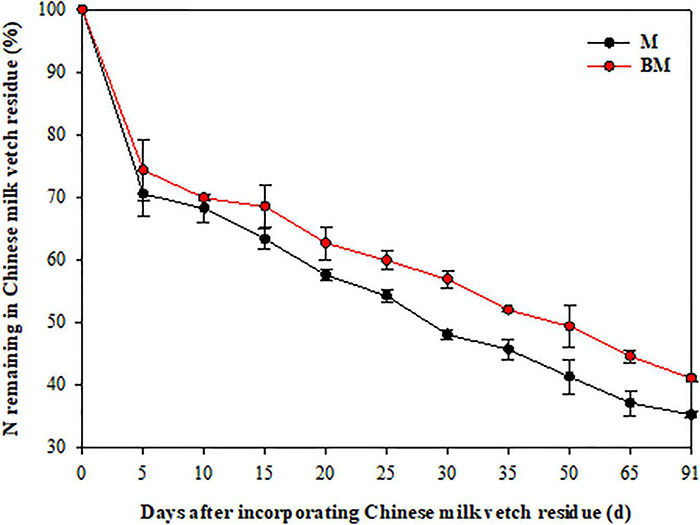
Nitrogen release pattern of leguminous cover crop residue after incorporation to reddish paddy soil. Small bars are standard errors.

More than 40% of the mass was lost and 30% of N released in 15 days after LCC residue incorporation, and thereafter both these factors slowed down (Figures 1, 2). After 91 days, only 34.4 and 37.6% of the mass, and 35.2 and 41.0% of N remained in M and biochar and treatment (BM) pots, respectively.

As the two-step nature of decay dynamics, the first-order double exponential decay model was used to estimate rate constants (*k*) that describe the mass loss or N release from LCC residue after incorporation over time (*t*) (Table 1). The highest rates of mass decay (*k*_*D_1*_ = 0.6126) and N release (*k*_*N_1*_ = 0.3687) were found at relatively rapid decay or release step in BM. However, the highest mass decay rates (*k*_*D_2*_ = 0.0076) and highest N release rates (*k*_*N_2*_ = 0.0101) were found at relatively slower mass degrading and N release steps of LCC residue in case of M alone treatment (Table 1).

**TABLE 1:** The rate constants (*k*) of mass decomposition and N release for leguminous cover crop residue with or without rice straw biochar estimated by using a double exponential model.

Treatment	Double exponential decay models	*k*_*1*_	*k*_*2*_	*R* ^2^
***Dry matter decomposition***				
M	D_M_ = 40.6e^–0.4973 t^+59.4e^–0.0076 t^	0.4973*	0.0076***	0.9879***
BM	D_BM_ = 41.0e^–0.6299 t^+59.0e^–0.0061 t^	0.6299*	0.0061***	0.9950***
***Nitrogen release***				
M	N_M_ = 29.6e^–0.3369 t^ +70.2e^–0.0101 t^	0.3369^+^	0.0101***	0.9802***
BM	N_BM_ = 26.3e^–0.3687 t^+73.7e^–0.0079 t^	0.3687*	0.0079***	0.9886***

The abbreviations M stand for legume cover crop while BM stand for a combination of both legume cover crop and rice straw biochar, and “+” means marginal significant (P = 0.0575), *P < 0.05, ***P < 0.001.

Compared to LCC residue alone, the addition of RSB slightly stimulated the rates of mass decomposition and N release from LCC residue after incorporation into paddy soil, while it notably slowed down the rates of mass decaying and N release from LCC residue for 15 days after incorporation in case of BM treatment at relatively slower stage (Table 1 and Figures 1, 2).

### Dry Matter Weight and Yield Components of Rice Plants

Incorporating RSB and/or LCC residue improved yield components and shoot biomass (Table 2). For instance, compared to 100% N, applying 80% N plus combination of RSB and LCC (N_80_BM) increased the 1,000-grain weight and filled grains per panicle (*p* > 0.05). Similarly, the number of effective panicles per plant was also markedly increased by 6.36%. Consequently, 7.11% (*p* < 0.05) increase in grain yield was observed. Almost similar and at par trends were observed for N_80_B and N_80_M treatments. However, additional RSB slightly suppressed the yield-increasing effect of LCC residue as green manure on rice plants mainly *via* decreasing the 1,000-grain weight (by ∼3.81%) and filled grains per panicle (by ∼1.25%).

**TABLE 2:** Effects of applying rice straw biochar and/or leguminous cover crop residue on dry matter weight and yield components of rice plants.

Treatment	1000-grain weight (g)	Effective panicle (No. /plant)	Full-filled grains (No. /panicle)	Dry matter weight (g/plant)		
				Straw	Grain	Shoot
CK	20.0 ± 0.75c	7.30 ± 0.58c	101.7 ± 6.77b	14.4 ± 0.54b	12.3 ± 0.87c	26.6 ± 1.37c
N_100_	21.9 ± 0.15b	11.0 ± 0.01b	108.5 ± 3.15a	20.8 ± 0.51a	21.1 ± 1.25b	42.0 ± 1.68b
N_80_M	23.6 ± 0.44a	11.3 ± 0.56ab	112.0 ± 3.92a	21.6 ± 0.57a	23.6 ± 0.44a	45.2 ± 0.35a
N_80_B	21.9 ± 0.83b	11.0 ± 1.00ab	109.3 ± 8.60a	21.1 ± 0.70a	21.7 ± 0.73b	42.8 ± 1.53b
N_80_BM	22.7 ± 0.97ab	11.7 ± 0.49a	110.6 ± 4.18a	20.8 ± 0.43a	22.6 ± 0.87a	43.4 ± 1.22ab
*F*-values	10.7**	23.6**	4.73*	87.5**	94.3**	116.2**

The abbreviations CK stand for no N fertilizer, rice straw biochar or legume crop cover crop, N_100_ for 150 Kg N ha^–1^, N_80_M for 80% N applied in conjunction with legume cover crop, N_80_B for 80% N applied in conjunction with rice straw biochar, and N_80_BM for 80% N applied in conjunction with both legume cover crop and rice straw biochar. Data were expressed as mean ± standard error (n = 3). Values followed by different letters in the same column are significantly different at P < 0.05. F-values marked with * and ** are significant at 0.05 and 0.01, respectively.

### Accumulation of N in Rice Plant Shoot

**FIGURE 3:**
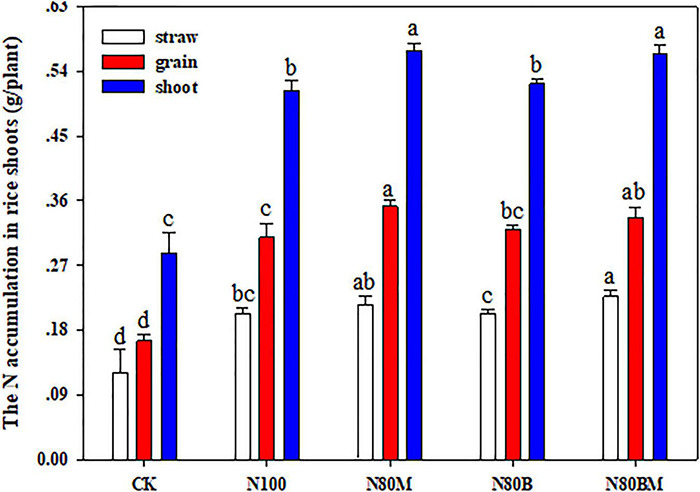
Effects of applying rice straw biochar and leguminous cover crop residue on the N accumulation in rice plant shoot at maturing stage. Small bars are standard errors. Values followed by different letters in the same color are significantly different at *P <* 0.05.

Understandably, all the fertilization treatments remarkably improved the N accumulation in rice straw and grain relative to unfertilized treatment (Figure 3). In case of applying 80% N, N accumulation in rice straw and grain did not significantly increase in N_80_B but applying LCC residue alone or in combination with RSB as N_80_M or N_80_BM resulted in markedly (*p* < 0.05) higher N accumulation in grain (by ∼13.87 and 8.96%), which eventually caused a noticeable increase in the shoot (by ∼10.09 and 10.91%), when compared with N_100_ treatment, respectively (Figure 3).

### Physiological N Use Efficiency of Rice Plants

Though *PE*_*N*_ did not prominently change in N_80_M pots, it was remarkably increased in the straw (by ∼28.8 and 6.80%) and grain (by ∼23.7 and 14.5%) and eventually caused a noticeable increase in the shoot of rice (by ∼25.3 and 9.75%) of N_80_B and N_80_BM pots when compared with N_100_, respectively (Table 3).

**TABLE 3:** Effects of applying rice straw biochar and/or leguminous cover crop residue on physiological N-use efficiency of rice after harvesting plants.

Treatment	Straw	Grain	Shoot
N_100_	79.4 ± 8.35c	61.2 ± 2.66c	67.7 ± 3.68c
N_80_M	76.3 ± 8.29c	60.5 ± 3.96c	65.8 ± 4.66c
N_80_B	102.3 ± 10.5a	75.7 ± 8.02a	84.8 ± 2.01a
N_80_BM	84.8 ± 7.21b	70.1 ± 4.49b	74.3 ± 5.35b
*F*-values	41.1**	8.19*	5.01*

The abbreviations CK stand for no N fertilizer, rice straw biochar or legume crop cover crop, N_100_ for 150 Kg N ha^–1^, N_80_M for 80% N applied in conjunction with legume cover crop, N_80_B for 80% N applied in conjunction with rice straw biochar, and N_80_BM for 80% N applied in conjunction with both legume cover crop and rice straw biochar. Data were expressed as mean ± SE (n = 3). Values followed by different letters in the same column are significantly different at P < 0.05. F-values marked with * and ** are significant at 0.05 and 0.01, respectively.

### Soil Chemical Properties

The contents of SOM and TN in plow layer of paddy soil after harvesting rice plants clearly showed higher values in N_80_M, N_80_B, and N_80_BM pots, revealing greater soil fertility and N-capacity under these treatments (Table 4). Under 80% N plus additional RSB and/or LCC residue (N_80_M, N_80_B, and N_80_BM treatments), higher SOM of 7.06–29.4% was observed than N_100_ pots. Similarly, the maximum TN content in soil was observed in N_80_M and N_80_BM treatments (1.00 and 0.92 g/kg, respectively) which was remarkably higher by 17.6 and 8.24% than N_100_, respectively.

**TABLE 4:** Effects of applying rice straw biochar and/or leguminous cover crop residue on the contents of soil organic matter (SOM), total N (TN), NH_4_^+^, and NO_3_^–^ after harvesting rice plants.

Treatment	SOM (g/kg)	TN (g/kg)	NH_4_^+^ (mg/kg)	NO_3_^–^ (mg/kg)
CK	14.3 ± 1.74d	0.72 ± 0.03d	3.18 ± 0.19d	0.67 ± 0.03e
N_100_	17.0 ± 0.03c	0.85 ± 0.06c	4.92 ± 0.06b	1.05 ± 0.04a
N_80_M	22.0 ± 1.47a	1.00 ± 0.05a	5.19 ± 0.12a	0.91 ± 0.03b
N_80_B	18.2 ± 2.60bc	0.87 ± 0.04bc	4.45 ± 0.09c	0.78 ± 0.01d
N_80_BM	20.9 ± 1.21ab	0.92 ± 0.05ab	4.74 ± 0.05b	0.84 ± 0.02c
*F*-values	10.6**	6.54*	40.4**	75.1**

The abbreviations CK stand for no N fertilizer, rice straw biochar or legume crop cover crop, N_100_ for 150 Kg N ha^–1^, N_80_M for 80% N applied in conjunction with legume cover crop, N_80_B for 80% N applied in conjunction with rice straw biochar, and N_80_BM for 80% N applied in conjunction with both legume cover crop and rice straw biochar. Data were expressed as mean ± SE (n = 3). Values followed by different letters in the same column are significantly different at P < 0.05. F-values marked with * and ** are significant at 0.05 and 0.01, respectively.

Compared to N_100_, 80% N in conjunction with LCC-only (N_80_M) significantly increased NH_4_^+^ content by 5.49% while decreasing NO_3_^–^ content by 13.3% (Table 4). Conversely, 80% N plus additional RSB and/or LCC residue (N_80_B and N_80_BM) notably decreased NH_4_^+^ content by 9.55 and 3.66%, and NO_3_^–^ content by 25.7 and 20.0% in comparison with N_100_, respectively (Table 4).

### Soil Microbial Population and Biomasses of C and N

**FIGURE 4:**
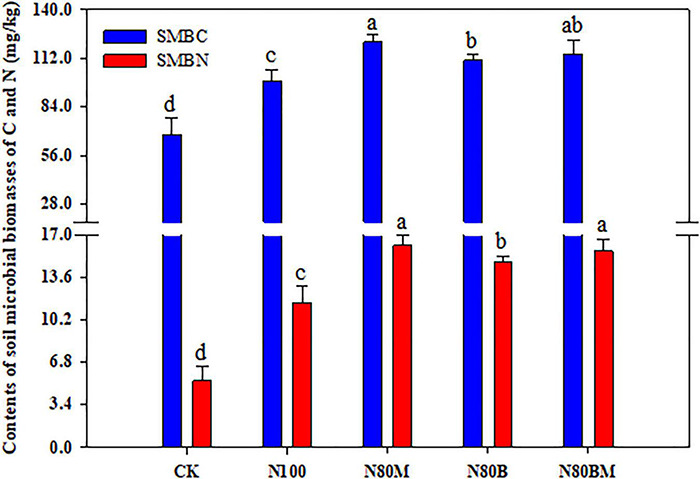
Effects of applying rice straw biochar and leguminous cover crop residue on soil microbial biomasses of C (SMBC) and N (SMBN) after harvesting rice plants. Small bars are standard errors. Values followed by different letters in the same color columns are significantly different at *P* < 0.05.

Additional RSB and/or LCC residue noticeably increased the SMBC and SMBN as compared to N_100_ (Figure 4). The obtained data illustrated that the paddy soil showed about 22.9, 12.5, and 16.3% increase in SMBC contents under RSB and/or LCC residue, respectively, when compared to N_100_. The SMBN contents in N_80_M, N_80_B, and N_80_BM were higher by 39.6, 28.0, and 35.6%, respectively, than N_100_.

## Discussion

### Mass Decomposition and N Release From Leguminous Cover Crop Residues After Incorporation Into Paddy Soil

Organic residues’ decomposition determines the soil nutrients pool by the way of de-polymerization of fibers and hydrolysis of sugars, which is mainly driven by the heterotrophic soil microbes, for nutrients cycling in agroecosystems (Meisinger and Ricigliano, 2017). A series of studies have simulated organic residues decay by using a nylon mesh bagging method (Yan et al., 2019; Dhakal et al., 2020). Compared to the single exponential decay model (Zhu et al., 2014), it is better to use a double exponential function with two rate constants (*k*), which separated organic residue into the fast pool (e.g., amino acids) and relatively slow pool (e.g., lignin) (Berg, 1984). The initial rapid phase biomass decomposition and N release mainly occurred in the first 2 weeks after LCC residue incorporation to paddy soil (Table 1 and Figures 1, 2). A logical explanation could be that the non-structural and labile fractions of LCC residue decompose rapidly, which supplied available C and nutrients for soil microorganisms, ultimately leading to increased microbial biomasses and their activities during the rapid phase of decomposition (Zhou et al., 2020). In addition, the availability of organic C plays an important role in influencing the decay kinetics (Walela et al., 2014). The simple fractions of LCC residue are gradually decreased over time, while the recalcitrant fractions are hard to be used by soil microbes that finally led to a relatively slower rate of mass decomposition and N release from LCC residue.

Moreover, the addition of RSB did not remarkably affect the rate constants of biomass decay and N release from LCC residue in the first 2 weeks after incorporation, though it significantly slowed down the rate constants (*k*_*2*_) at the relatively slower stage in paddy soil (Table 1 and Figures 1, 2). These findings are in line with the results obtained by Cheng et al. (2017). It can be thus inferred that RSB with a high C/N ratio (87.8) might have constrained mass decomposition and N release from incorporated LCC residue with a low C/N ratio (12.4) at the slower stage. The high C-rich and porous biochar would cause shifts in soil microbial properties (e.g., size, activity, and structure) that alter microbial C use efficiency, leading to greater partitioning of substrate C into catabolic processes. Thus, microbes will prefer to act on the most energetically advantageous organic matter and stimulate the decomposition of simple fractions at the early stage. Meanwhile, the complex compounds that incur higher energy cost for degradation would be left till the relatively slower stage in soil (Cheng et al., 2017). In addition, both the available C and N in organic amendments and soil help the soil microbes in biomass decaying during the initial rapid phase of decomposition, while the addition of RSB remarkably decreased inorganic N (NH_4_^+^ and NO_3_^–^) contents in paddy soil after rice harvest (Table 4), which was one of the main factors that might have constrained the microbial activity, and in turn influenced the decomposition and N release from LCC residue at the relatively slower stage (Zhang et al., 2015).

### Rice Yield and Its Components, Nitrogen Absorption, and Use Efficiency

Cover crops not only cover fallow soils but also contribute to producing more food without an increase in the use of chemical fertilizer (Xie et al., 2016; Yang L. et al., 2019). This is particularly true for LCC residues that provide N through biological N fixation and increase soil N supply to subsequent crops. In addition, biochar decreases the bulk density, increases electrical conductivity, available phosphorous, porosity, and water-stable aggregation (especially the macro-aggregates), and facilitates the retention of SOC and N nutrient in the soil, ensuring better conditions for plant growth, and thereby, promoting root growth and nutrients absorption of rice plants (Gao et al., 2019; Nan et al., 2020; Zhang et al., 2020; Kimani et al., 2021; Pandey et al., 2021). Application of RSB and/or LCC residue increased dry matter accumulation, N absorption, and *PE*_*N*_ of rice shoot when compared with 100% N alone (Tables 2, 3 and Figure 3), which were consistent with the results of Wu et al. (2019) and Zhou et al. (2020). This can be explained in the light of the following four phenomena, i.e., (i) LCC residue as green manure (GM) might increase enzymatic activities (e.g., that of Superoxide Dismutase, Catalase, and Nitrate Reductase) and decrease the Malondialdehyde content in rice root (Xie et al., 2017). Meanwhile, the addition of RSB accelerates ammonia oxidation and increases root activity (Cao et al., 2019). Thus, a combined application of RSB with LCC residue would synergistically promote N uptake and use efficiency; (ii) the yield components of rice crop (i.e., effective panicles, number of full-filled grains per panicle, and 1,000-grain weight) were significantly improved in LCC and/or RSB amended pots (Table 2). Earlier, Huang et al. (2013) reported that spikelet number per panicle and grain weight exhibited positive responses to crop residue retention. Similarly, improvements in leaf area index, photosynthetic rate, and ratio of photosynthetic potential to grain have been found under the application of organic materials in addition to better root growth and development of rice plants (Zhang et al., 2020). These further strengthened the supporting system and provided enough dry matter for yield formation and nutrient absorption by rice plants. Thus, the increase in grain yield under RSB and/or LCC application could be attributed to the more vigorous plant growth at the later stage that eventually led to greater sink capacity and guaranteed the grain filling of rice plants as has also been reported by Takai et al. (2006); (iii) application of RSB and/or LCC improved soil structure such that a better microenvironment near the rhizosphere was ensured resulting in higher density, biomass, and activity of rice root that in turn facilitated N uptake and use efficiency (Zhang et al., 2020); (iv) the combined application of organic amendments with different C/N ratios regulated N release rate that could better meet the N needs of rice plants and promote N absorption and use efficiency (Zhou et al., 2020). However, LCC residue had a larger increasing effect on early rice than RSB (Table 2). It can be ascribed to the lower C/N ratio of LCC residue that probably led to rapid decomposition and N release. Conversely, RSB with a higher C/N ratio notably restricted N release from LCC residue during the relatively slower N release stage (Table 1 and Figure 2).

### Soil Chemical Properties

Due to the intrinsic properties (i.e., larger surface area, micropores, and elemental compositions), the addition of biochar potentially alters the physical and chemical properties of the disturbed paddy soil (Chhatarpal et al., 2018). In comparison with 100% FN treatment, 80% N plus RSB with or without LCC residue increased the contents of SOM in soil (Table 4), which is in accordance with other studies (Yang S. H. et al., 2019; Zhang et al., 2020; Zhou et al., 2020). The increase in soil TN content could be linked to additional N from RSB (6.13 g/kg) or LCC residue (32.6 g kg^−1^) which was subsequently released into the soil (Song et al., 2018). In contrast, with regard to the increase in SOC, biochar could sequester native soil organic matter in its network of pores, thus reducing the degradability and mineralization of native organic matter in soil *via* microbial disintegration (Dungait et al., 2012). Moreover, biochar is rich in organic carbon that could form aggregates with soil particles that protect SOC from being decomposed (Amoakwah et al., 2020). Finally, biochar particles might also be integrated into the soil matrix (Zhang et al., 2020), resulting in a direct increase in the SOC content.

Furthermore, under 20% FN treatment, sole application of LCC residue remarkably increased NH_4_^+^ content while decreasing NO_3_^–^ content in reddish paddy soil, further confirming the results of our previous study (Xie et al., 2016). Additional RSB with or without LCC decreased NH_4_^+^ and NO_3_^–^ contents when compared with 100% N pots (Table 4), which is in agreement with the findings of Gao et al. (2019) and Martos et al. (2020). The probable mechanisms could be as follows: (i) the reduced N might have led to less available inorganic N (NH_4_^+^ and NO_3_^–^); (ii) addition of RSB and LCC facilitated N uptake and assimilation by NH_4_^+^-preferring rice plants (Figure 3) that further resulted in reducing available N substrates for nitrification and then limiting the nitrification of NH_4_^+^ to NO_3_^–^ (Yang et al., 2015); (iii) addition of RSB could obtain a remarkable increase in the relative abundance of nitrifiers which in turn accelerate ammonia oxidation (Nguyen et al., 2018), and meanwhile, promote NO_3_^–^ consumption by enhancing root activity and nitrate reductase activity in the roots of rice plants (Fungo et al., 2019; Sun et al., 2019); and (iv) the application of LCC residue as GM might mitigate the reduction in pH or even increase it (Li et al., 2019; Cheng et al., 2021). Conversely, the biochar elevated pH in acidic soil (Kimani et al., 2021), that in turn sharply regulated the forms of NO_3_^–^ and NH_4_^+^ and promoted ammonia volatilization from soil (Hailegnaw et al., 2019; Schofield et al., 2019), that could be captured in micropores or adsorbed to the inner and exterior large oxygen-containing surface of functional groups of biochar particles (Li et al., 2018); (v) the labile C from biochar made from crop residues might induce a relatively N-limited condition and enhance microbial demand for soil N to use additional degradable C (Lehmann et al., 2003), and ultimately decrease soil inorganic N (NH_4_^+^ and NO_3_^–^). In our study, RSB with or without LCC increased MBC and MBN contents in paddy soil (Figure 4). Interestingly, the combined application of RSB and LCC residue could offset the negative impact of only RSB addition on soil inorganic N to some extent (Table 4). This might have occurred due to the net gain of N that was caused by LCC residue as GM (Xie et al., 2016); and the extra organic N input may be probably preserved by forming a biochar-organo-mineral complex which can further contribute to mineralized N in soil (Zhang et al., 2020; Yang et al., 2021). These results demonstrate that the combination of RSB with LCC residue can be a novel and promising strategy for managing rice straw and N that can effectively mitigate the agro-environmental risks associated with reddish flooded paddy fields.

### Microbial Biomass of Carbon and Microbial Biomass of Nitrogen in Paddy Soil

Generally, the MBC and MBN pools in soil are regarded as suitable indicators of soil health and functions according to different fertilization strategies. Our findings indicate that reducing 20% N when coupled with the addition of RSB and/or LCC residue as GM notably increased microbial biomass C and N in paddy soil when compared with 100% N (Figure 4). Applying organic amendments (e.g., biochar and/or crop residues) increases microbial biomasses in acidic soils even under low fertility (Pokharel et al., 2020; Zhou et al., 2020). Moreover, soil pH and SOC are the main factors regulating the activities of soil enzymes. Compared with sole application of chemical fertilizers, applying organic amendments would have more positive influences on microbial biomasses as is evident from the considerable increases of SMBC and SMBN contents, soil pH, C and N dynamics, and the activities of C- and N-cycle-related enzymes that can stimulate soil microbial growth (Luo et al., 2018; Zhou et al., 2020).

## Conclusion

Results of the two pot experiments showed that the addition of RSB promoted the rates of mass decomposition and N release from LCC residue after incorporation into paddy soil to some extent during the initial rapid stage of decomposition, and distinctly suppressed their rates during the relatively slower stage. A 20% reduced N application when coupled with RSB and/or LCC residue addition improved rice yield components and increased soil fertility that subsequently led to higher grain and straw yields, and N absorption and use efficiency in rice shoots. However, the addition of RSB with or without LCC residues decreased NH_4_^+^ and NO_3_^–^ contents in the reddish paddy soil. Therefore, it seems logical to conclude that combining RSB with LCC residue can be a novel and promising intervention for improving soil fertility and productivity while decreasing N pollution caused by N losses in rice-based cropping systems. Future studies should further focus on the long-term and field-scale effects of combining RSB and LCC residue and their overall impact on agro-environmental sustainability of the reddish paddy ecosystems of south China. However, field-scale trials are imperative in the near future to confirm and ascertain the long-term benefits of these organic amendments.

## Data Availability Statement

The original contributions presented in the study are included in the article/supplementary material, further inquiries can be directed to the corresponding author.

## Author Contributions

ZX conceived and performed the experiment and drafted the manuscript. FS participated in the manuscript preparation. CZ provided the facilities. All authors contributed to the article and approved the submitted version.

## Conflict of Interest

The authors declare that the research was conducted in the absence of any commercial or financial relationships that could be construed as a potential conflict of interest.

## Publisher’s Note

All claims expressed in this article are solely those of the authors and do not necessarily represent those of their affiliated organizations, or those of the publisher, the editors and the reviewers. Any product that may be evaluated in this article, or claim that may be made by its manufacturer, is not guaranteed or endorsed by the publisher.
